# Validation of case definitions of depression derived from administrative data against the CIDI-SF as reference standard: results from the PROspective Québec (PROQ) study

**DOI:** 10.1186/s12888-021-03501-x

**Published:** 2021-10-07

**Authors:** Ana Paula Bruno Pena-Gralle, Denis Talbot, Xavier Trudel, Karine Aubé, Alain Lesage, Sophie Lauzier, Alain Milot, Chantal Brisson

**Affiliations:** 1 CHU de Québec Research Center, Population Health and Optimal Health Practices Unit, Québec, QC Canada; 2 Faculty of Medicine, Laval University, Québec, QC Canada; 3 Département de Psychiatrie et d’addictologie, Université de Montréal, Montréal, Canada; 4 Faculty of Pharmacy, Laval University, Québec, QC Canada; 5 Centre de recherche sur les soins et les services de première ligne de l’Université Laval, Québec, QC Canada

**Keywords:** Cohort, Population-based, Major depression, known-groups analysis, Known-groups validity, Predictive validity

## Abstract

**Background:**

Administrative data have several advantages over questionnaire and interview data to identify cases of depression: they are usually inexpensive, available for a long period of time and are less subject to recall bias and differential classification errors. However, the validity of administrative data in the correct identification of depression has not yet been studied in general populations. The present study aimed to 1) evaluate the sensitivity and specificity of administrative cases of depression using the validated Composite International Diagnostic Interview – Short Form (CIDI-SF) as reference standard and 2) compare the known-groups validity between administrative and CIDI-SF cases of depression.

**Methods:**

The 5487 participants seen at the last wave (2015–2018) of the PROQ cohort had CIDI-SF questionnaire data linked to hospitalization and medical reimbursement data provided by the provincial universal healthcare provider and coded using the International Classification of Disease. We analyzed the sensitivity and specificity of several case definitions of depression from this administrative data. Their association with known predictors of depression was estimated using robust Poisson regression models.

**Results:**

Administrative cases of depression showed high specificity (≥ 96%), low sensitivity (19–32%), and rather low agreement (Cohen’s kappa of 0.21–0.25) compared with the CIDI-SF. These results were consistent over strata of sex, age and education level and with varying case definitions. In known-groups analysis, the administrative cases of depression were comparable to that of CIDI-SF cases (RR for sex: 1.80 vs 2.03 respectively, age: 1.53 vs 1.40, education: 1.52 vs 1.28, psychological distress: 2.21 vs 2.65).

**Conclusion:**

The results obtained in this large sample of a general population suggest that the dimensions of depression captured by administrative data and by the CIDI-SF are partially distinct. However, their known-groups validity in relation to risk factors for depression was similar to that of CIDI-SF cases. We suggest that neither of these data sources is superior to the other in the context of large epidemiological studies aiming to identify and quantify risk factors for depression.

**Supplementary Information:**

The online version contains supplementary material available at 10.1186/s12888-021-03501-x.

## Introduction

Depression is the most common mental disorder worldwide: according to estimates of the World Health Organization (WHO), the 12-month prevalence of depression is 4.4% [1] while the lifetime prevalence is 10% [2–4]. Depression is the third most important cause of disability-adjusted years of life, with trend growth for leading cause by 2030 [5]. Additionally, recent studies have observed a positive association between depression and the onset and/or worsening of chronic diseases such as diabetes [6], cardiovascular diseases [7] and dementias [8].

Cases of depression can be estimated from studies that employed clinical interviews or questionnaires, or from administrative data such as physician billing or hospital discharge data [9]. The latter have several advantages over questionnaires: they are usually inexpensive, available for a long period of time and are less subject to recall bias and differential misclassification. Additionally, administrative data generally observe international standardization through the WHO International Classification of Diseases and Health Problems (ICD) codes, classified in the ICD-9 and ICD-10 systems [10]. However, two important obstacles to their large-scale use need to be considered: 1) misclassification in the detection of depression at the primary care level [11] and among general practitioners [12, 13]; and 2) a possible underestimation of cases, given that only those who actively sought help from a medical professional are counted.

Previous studies have attempted to evaluate the validity of depression cases obtained from administrative data [9, 14–22], but most of them used samples defined by pre-existing comorbidities [20], such as inflammatory bowel disease [9], rheumatism [21] or diabetes [22], or samples of patients who had sought ambulant care [14, 15] or were hospitalized [16–19]. In these studies, the sensitivity of administrative data in detecting cases of depression was sub-optimal: 29–36% [17], 50% [9], and 61% [16], which is important when evaluating the suitability of administrative data for classifying individuals as cases. However, all the populations studied are known to be more vulnerable to depression. If one is interested in the effects of risk factors or of preventive interventions on its incidence, it would therefore not be possible to extrapolate results from such samples to the general population.

In contrast with existing data, the present study was conducted in a diverse cohort of active and retired workers from the general population, without restriction to those who had sought medical help or those with a pre-existing disease. The objectives were: 1) to evaluate the sensitivity and specificity of cases of depression detected using administrative data compared with cases identified using the Composite International Diagnostic Interview – Short Form (CIDI-SF; a validated instrument to detect depression) as reference standard; and 2) to test the suitability of administrative cases of depression for association studies by estimating the effects of sex, age, educational level and psychological distress and comparing them with the effects estimated from CIDI-SF cases.

## Methods

### Study design and population

The PROspective Québec (PROQ) study is a prospective cohort that has followed 9188 white-collar workers (49.8% women) since 1991 in Quebec, Canada [23]. Questionnaires, clinical measurements and interviews were used to collect participants’ data (socioeconomic conditions, lifestyle habits and physical and psychological health) at three data collection waves: 1991–1993 (*n* = 9188), 1999–2001 (*n* = 8120) and 2015–2018 (*n* = 6744). For the present study, the sample was restricted to participants who responded to the CIDI-SF instrument at the last data collection wave and who consented to having their data linked with the administrative data provided by the provincial universal healthcare provider, the *Régie de l’Assurance Maladie du Québec* (RAMQ; *n* = 5487, 66% of those eligible to this last wave).

### CIDI-SF cases of depression

Cases of depression were estimated in 2015–18 using a validated French version of the WHO’s Composite International Diagnostic Interview-Short Form (CIDI-SF) [24, 25]. CIDI-SF is consistent with the definition of depression in the Diagnostic and Statistical Manual of Mental Disorders (DSM-IV) and, more broadly, with that in the International Classification of Diseases (ICD-10). Following DSM-IV, participants were considered cases if they reported having had at least five depressive symptoms almost all day, almost every day, for a period of two consecutive weeks or more in the past 12 months. The symptoms were 1) depressed mood (dysphoria), 2) loss of interest in most things (anhedonia), 3) fatigue / lack of energy, 4) weight change (loss or gain), 5) difficulty falling asleep, 6) difficulty concentrating, 7) feeling down or worthless, and 8) thinking about death, one’s own or that of others. Dysphoria or anhedonia had to be present among the five symptoms [26]. Validation studies of this instrument observed a > 90% probability of detecting depression when applying this definition [27, 28].

### Administrative cases of depression

RAMQ provides almost all health services in Quebec [29]. All administrative data was extracted from three RAMQ databases (population registry, hospital discharge abstracts and physician services) for the period from January 1, 1991 to December 31, 2018. Hospital admission and discharge dates and ICD codes were obtained from the hospital discharge abstracts (ICD-9 before 2006, ICD-10 beginning in 2006). Consultation dates associated with ICD-9 codes for ambulant treatment were extracted from the physician services database. For the main analysis, we considered ICD-9 codes 296.x, 300.4, 309.x and 311.x and ICD-10 codes F30-F34, F39 and F43.2. To estimate incidence, dates of death were also obtained from the population registry. These databases were joined with each other and with the PROQ cohort database using unique personal identification codes.

Based on previous investigations [9, 16, 17], we considered any hospital stay associated with a diagnosis of depression sufficient to constitute an administrative case, but evaluated two different case definitions with regard to medical consultations:

A) Two consultations on different days during 1 year leading to the diagnosis of depression.
B) Any consultation leading to a diagnosis of depression.

For the purpose of comparing with CIDI-SF cases (which referred to the year preceding the last wave), the case definitions of administrative cases were applied to that same year.

### Known risk factors for depression

Known risk factors for depression were used to assess known-groups validity, which is established when an instrument can effectively distinguish between two groups who are known a priori to differ on the variable of interest [30, 31]. In this case, we used sociodemographic variables that are known risk factors for depression: sex (man/woman); age (dichotomized at the median age at retirement) and level of education at baseline (with or without a university degree). In addition, we defined groups based on psychological distress, measured in 1999–2001 using the validated French version of the Psychiatric Symptom Index [32, 33]. Scores were dichotomized at the median.

### Analysis

Sensitivity, specificity, positive predictive value (PPV), negative predictive value (NPV), and likelihood ratios (LR+, LR-) each with its 95% confidence interval (95% CI), were calculated for each case definition of depression derived from administrative data, using the CIDI-SF as reference standard. We also calculated Cohen’s kappa (κ) [34].

Furthermore, additional analyses were carried out in order to compensate for possible effects of selective attrition during the follow-up. We used multiple imputation by chained eqs. (20 imputations, 10 iterations) first to infer the missing data from the covariates obtained at recruitment (< 5% missing data, 1991–1993), and then the missing data from the second wave (< 10% missing data, 1999–2003). Using the multiply imputed data, we adjusted the prevalence of depression at the third wave (2015–2018) by inverse probability of censoring weighting (IPCW), which gives more weight to the participants that are more similar to the ones that were lost to follow-up, and therefore permits inferring a population without attrition. The probability of censoring of each subject was estimated as the predicted value of a logistic regression where the dependent variable was whether the subject was censored, and the independent variables were sociodemographic ones [sex, age (continuous), marital status (married or living together vs. living alone), and health behaviors [smoking (never, in the past, currently), alcohol consumption (≤ 5 doses/week, > 5 doses /week), physical activity (≤ once/month, ≤ once/week, ≥ once/week) and body-mass index (weight (kg) by square of height (m))], measured at the first two waves. Sensitivity, specificity, PPV, NPV and kappa with their 95% CI were estimated from the imputed and weighted datasets using non-parametric bootstrap (2000 replications) [35].

For the known-groups analysis, we estimated the prevalence of depression separately for each stratum of four known risk factors for depression: sex, age, educational level and psychological distress [32]. To quantify the relative risk, the association between each risk factor and each case definition of depression was estimated using a robust Poisson model. This is a convenient approach to estimate prevalence ratios with a binary outcome while being less sensitive to model misspecification and avoiding the non-convergence problems of the log-binomial regression [36]. The analyses were conducted in software R, version 3.6.1 with the *tidyverse*, *epiR*, *geepack*, *mice* [37] and *boot* [38] packages.

## Results

**Fig. 1:**
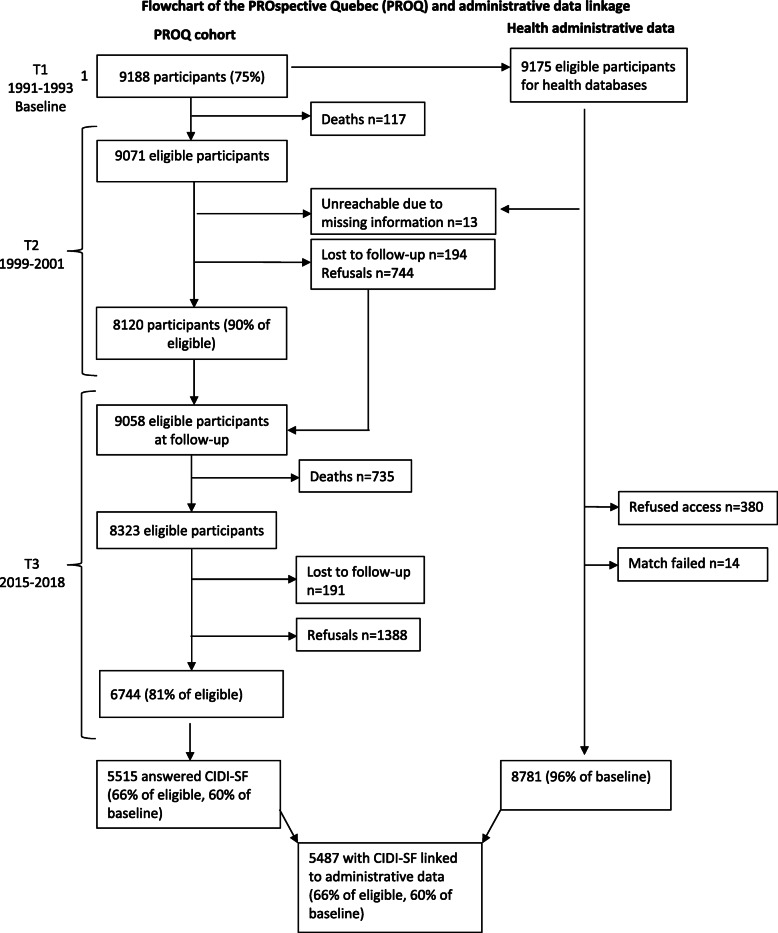
Flowchart of the PROspective Quebec (PROQ) and administrative data linkage

Among the initial 9188 participants, 8781 (95.6%) consented to give access to their healthcare administrative data. By the last data collection wave, 852 participants had died. Among the 6744 that participated in the last wave, 5515 answered the CIDI-SF questionnaire (Fig. 1). Table 1 shows the distribution of the participants’ characteristics at each wave of the study and among the respondents to the CIDI-SF. The final sample was similar to the entire participating population at that wave and comparable to the baseline one.

**Table 1:** Cohort characteristics at each of the three data collection waves and characteristics of CIDI-SF respondents

Characteristics	T_**1**_ ***n*** = 9188	T_**2**_ n = 8120	T_**3**_ ***n*** = 6748	CIDI-SF^a^ ***n*** = 5515
**Age**	40.0 ± 8.6 (19–72)	47.8 ± 8.5 (26–80)	63.7 ± 7.6 (44–98)	64.0 ± 7.2 (44–89)
**Sex**				
Women	4581 (49.9%)	4000 (49.3%)	3357 (49.8%)	2674 (48.5%)
Men	4607 (50.1%)	4120 (50.7%)	3391 (50.2%)	2841 (51.5%)
**Education**				
≤ High school	2715 (29.8%)	2170 (27.0%)	1641 (24.6%)	1342 (24.4%)
College	2564 (28.1%)	2233 (27.8%)	1787 (26.8%)	1454 (26.5%)
≥ University	3835 (42.1%)	3634 (45.2%)	3253 (48.7%)	2699 (49.1%)
**Administrative cases of depression**^b^			255 (3.8%)	207 (3.8%)

^a^ CIDI-SF respondent are a subset of all participants in the T_3_ data collection wave. ^b^ At least one depression code (hospital or ambulatory) during the year preceding T_3_

Both questionnaire and administrative data were available for 5487 participants (Fig. 1). In this population, the one-year prevalence of CIDI-SF cases of depression was 4.0% (221 participants); in women, it was two times higher than in men (5.5% vs. 2.7%). For this same year, the prevalence of administrative cases was 2.4% (125 participants) using the more restrictive case definition (A) and 3.8% (207 participants) using the less constrained case definition (B). All depression-related codes in our population were given by general practitioners or psychiatrists.

Administrative cases of depression based on definition A had a sensitivity of 18.6% and a PPV of 32.8%. Concordance with CIDI-SF cases was reasonable (κ = 0.214, Table 2). On the other hand, definition B had a higher sensitivity (24.0%) and somewhat lower PPV (25.6%); the overall concordance with CIDI-SF cases was very similar as for definition A (κ = 0.217). Specificity and NPV were very high for both comparisons (Table 2). In order to exclude possible effects of missing cohort data and attrition on these estimates, we also performed multiple imputation followed by inverse probability of censoring weighting. Sensitivity, PPV and concordance were all slightly higher than for the complete cases (Table 2).

**Table 2:** Agreement between administrative cases and CIDI-SF cases of depression at T_3_ (2015–2018)

Definition of administrative cases of depression	Sensitivity (CI 95%)	Specificity (CI 95%)	PPV (CI 95%)	NPV (CI 95%)	κ (CI 95%)	LR+(CI 95%)	LR-(CI 95%)
Definition A^a^ (complete cases)	18.6 (13.7–24.3)	98.4 (98.0–98.7)	32.8 (24.7–41.8)	96.6 (96.1–97.1)	.214(.154–.274)	11.6 (8.2–16.5)	.828(.777–.882)
Definition A^a^ (MI followed by IPCW)	19.6 (13.9–25.2)	98.2 (97.8–98.6)	32.1 (23.4–40.9)	96.6 (96.1–97.1)	.219 (.153–.284)	10.9 (6.8–15.0)	.819(.761–.877)
Definition B^b^ (complete cases)	24.0 (18.5–30.2)	97.1 (96.6–97.5)	25.6 (19.8–32.1)	96.8 (96.3–97.3)	.217(.162–.273)	8.2 (6.2–10.9)	.783(.727–.843)
Definition B^b^ (MI followed by IPCW)	25.0 (19.1–30.9)	97.0 (96.5–97.4)	26.2 (20.1–32.3)	96.8 (96.3–97.3)	.225 (.167–.282)	8.3 (5.9–10.6)	.773(.713–.834)

Reference CIDI-SF. *MI* multiple imputation; *IPCW* inverse probability censoring weighted; *PPV* positive predictive value; *NPV* negative predictive value; *κ* Cohen’s kappa; *LR+* positive likelihood ratio; *LR-* negative likelihood ratio. ^a^ Any hospital stay or two medical consultations within the year preceding CIDI-SF. ^b^ Any hospital stay or medical consultation within the year preceding CIDI-SF

We also estimated the agreement between CIDI-SF and administrative cases of depression after stratifying for sex, age and education level. While prevalence was higher in women for all case definitions (Table 3), agreement between the two definitions of depression was not very different in the strata of sex, age and education (Suppl. Table 2).

**Table 3:** One-year prevalence of depression and known-groups analysis for sex, age, education level and psychological distress

	CIDI-SF cases			Administrative cases ^a^			Administrative cases ^b^		
	Prev.(non-exposed)	Prev.(exposed)	Ratio (IC95%)	Prev.(non-exposed)	Prev.(exposed)	Ratio (IC95%)	Prev.(non-exposed)	Prev.(exposed)	Ratio (IC95%)
**Total**	4.03%			2.37%			3.77%		
**Sex (Ref. male)**	2.68%	5.46%	2.03 1.55–2.66	1.64%	3.11%	1.901.43–2.52	2.72%	4.89%	1.801.36–2.37
**Age (Ref. > 58 years old**	4.08%	5.96%	1.401.05–1.87	2.00%	3.45%	1.721.25–2.37	3.39%	5.20%	1.531.15–2.05
**Education T**_**1**_ **(Ref. univ. degree)**	3.46%	4.48%	1.28 0.98–1.67	1.65%	2.91%	1.761.31–2.37	2.96%	4.50%	1.521.15–2.01
**Psychological distress T**_**2**_ **(Ref. < 26.2)**	2.90%	7.69%	2.65 2.03–3.47	1.78%	3.87%	2.171.62–2.91	2.97%	6.56%	2.211.67–2.92

^a^ Definition A of administrative cases: any hospital stay or two medical consultations within the year

^b^ Definition B of administrative cases: any hospital stay or medical consultation within the year

In order to further test the robustness of these estimates, we also performed eight supplementary analyses where we either broadened or restricted the algorithms and criteria for CIDI-SF and administrative cases of depression (Suppl. Table 3). We tested longer periods of time for the occurrence of diagnoses (18 or 24 months) and lowered the threshold of depression for the CIDI-SF. We also restricted the ICD-9 and ICD-10 codes to exclude ambiguous cases (e.g. co-occurrence of depression with anxiety; Suppl. Table 4) or to diagnoses made by psychiatrists.

We noted that among the service claims made by general practitioners and psychiatrists during the years of interest for the participants not known to be administrative cases of depression, 16.0% did not contain any ICD code. It is possible that some proportion of these claims should have been coded as due to depression. Therefore, we also analysed a population excluding all controls with any missing ICD code (Suppl. Table 4). None of these alternative case definitions increased the concordance considerably.

In order to evaluate known-groups validity, we tested the association of sex, age, educational level and psychological distress with our two case definitions of administrative cases and, for comparison, with CIDI-SF cases of depression (Table 3). In univariate analyses, women, participants without a university degree and those aged 58 years or less had higher risks of being both CIDI-SF cases (as expected [1, 39]) and administrative cases. In fact, administrative cases of depression (definition B) discriminated all three factors to a similar degree as did CIDI-SF cases (RR for sex: 1.80 vs 2.03 respectively, age: 1.53 vs 1.40; education: 1.52 vs 1.28). The associations of known risk factors with definition A of administrative cases were slightly but consistently larger than with definition B (RR for sex: 1.90, age: 1.72; education: 1.76).

We also assessed known-groups validity using groups defined by level of psychological distress measured in the second wave of data collection. The administrative definitions of depression were able to discriminate between groups of higher and lower distress (RR = 2.17 and 2.21), though discrimination by CIDI-SF seemed slightly higher (RR = 2.65; Table 3).

Finally, we assessed known-groups validity after excluding all controls with any missing ICD code. The relative risks are almost identical to the ones observed in the main analysis (Table 3; Suppl. Table 5).

## Discussion

This study evaluated the validity of cases of depression detected using administrative data compared with cases identified using the CIDI-SF as reference. High specificity, but a low sensitivity and rather low agreement were observed. However, administrative cases of depression performed as well as CIDI-SF cases in discriminating risk groups based on sex, age, educational level and psychological distress (Table 3). To our knowledge, this is the first study that investigates the validity of different case definitions of depression derived from administrative data in a general population of active and retired workers.

Altogether we tested the validity of ten case definitions of depression based on administrative data. None of these definitions had an agreement with CIDI-SF of more than 0.25 (Table 2, Suppl. Table 3). A PPV of 33% for administrative cases (Table 2) means that 67% of the participants who were diagnosed with depression by a physician during the previous year were not identified as CIDI-SF cases. This suggests that administrative data may capture different dimensions of depression compared to the questionnaire data.

The prevalence of depression in our cohort, estimated using CIDI-SF, is similar to the one estimated for the general Canadian population using the more complete World Mental Health-CIDI-questionnaire (4.0% vs. 4.7% [40]). This makes it unlikely that the rather low agreement observed here is due to the way we defined cases from the CIDI-SF. In fact, it remained low even when using an extremely flexible threshold (Suppl. Table 3). Some hypotheses may be advanced to explain the discrepancy between administrative and questionnaire cases:

1. *Dynamic character of depression.* Given that depression is an episodic and recurrent condition, some participants may have been asymptomatic at data collection despite suffering from depression a few months earlier. Treatment may also have attenuated their symptoms or led to remission. The stigma associated with depression [41] might have predisposed them towards omitting their recent depressive episode while responding to the CIDI-SF. Furthermore, participants who consider that an episode of depression in the more distant past has already resolved, may still be considered as suffering from depression by health professionals [42].
2. *Overestimation of the one-year prevalence of depression by CIDF-SF.* CIDI-SF is not a perfect tool for diagnosing depression. CIDI-SF seems to overestimate the one-year prevalence of depression when compared to a structured clinical interview [28], and in some cases, though not apparently in our cohort, when compared to the longer World Mental Health-CIDI questionnaire [43]. Some of the CIDI-SF cases, even though they fulfil the DSM criteria for a major depression episode, might be viewed as subclinical in a consultation, and if CIDI-SF in fact overestimated depression prevalence, then the sensitivity of administrative cases may not be quite as low as we found.
3. *Underestimation of the incidence of depression by administrative data*. Administrative data only capture cases of depression where the patient actively sought out a medical professional. There is a social stigma surrounding depression, which associates the illness with characteristics such as weakness, inadequacy and lack of effort that may prevent many from seeking professional help [41]. Additionally, our administrative data are blind to cases of depression that were not treated by physicians or in hospitals. Thus, our case definitions exclude those who only sought treatment from clinical psychologists, social workers or other non-medical mental health professionals.
4. *Underestimation of the incidence of depression by low sensitivity of case detection.* Furthermore, results of two meta-analyses suggest that general practitioners identify less than half and maybe only a third of the cases recognized by specialists [44, 45]. In Quebec’s healthcare system, patients first consult with a family doctor, who is usually a general practitioner, before being referred to a specialist; this means that the incidence of administrative cases of depression might be underestimated. Even in a clinical population (hospitalized participants) and using complete medical records as the gold standard, case definitions from administrative data had relatively low sensitivities, ranging from 29 to 36% [17]. In a population of patients with inflammatory bowel disease, sensitivity was 50% and κ = 0.42, even though the prevalence of depression in this population was twice as high as in the general population [9]. One possible mechanism for underestimation is through missing data; in fact, in our population, for 16.0% of physician services claims no ICD code was filled out; some of the cases of depression not recognized by physicians might manifest as claims without any ICD code.

Nevertheless, in our study, administrative cases were as good at discriminating risk groups as CIDI-SF cases were, according to all case definitions used in this study. The association of the known risk factors with administrative cases might be put down to increased help-seeking. However, in this case, we would expect participants with lower levels of education to be underrepresented in our administrative data, while in fact they are overrepresented. Furthermore, the agreement of administrative and questionnaire cases is very similar in all strata (Suppl. Table 2). We therefore conclude that association with help-seeking is unlikely to entirely explain the association between classical risk factors and our administrative definitions of depression.

### Strengths and limitations

The strengths of this study include the use of a large cohort of workers, not restricted to those who had sought medical help or those with a pre-existing disease. Moreover, we had access to administrative data for 96% of the cohort. The high rates of participation at recruitment and of consent to access administrative data reduce a possible selection bias. Furthermore, the observed socio-demographical characteristics do not indicate selective attrition during follow-up, suggesting a low risk of selection bias for the CIDI-SF questionnaire data. The results obtained after multiple imputation and IPCW were similar to those found using complete cases, also reducing the possibility of selection bias. Finally, the size of the population was sufficient for estimating sensitivity, specificity, predictive values and concordance between administrative and questionnaire data in different socio-demographical strata and under a variety of case definitions.

Our study also has limitations. First, our population was composed of white-collar workers in Quebec’s public and semi-public sector and might not reflect the general population. However, the one-year prevalence of depression based on CIDI-SF cases found in this cohort is very similar to that obtained for the overall Canadian population [40]. Furthermore, while we used a questionnaire validated for diagnosing clinical cases of depression as reference, we recognize that the ideal reference measure would be the use of the Structured Clinical Interview for DSM-IV [28]. There are medical services claims without diagnostic code in the administrative data; however, two different approaches to treating these missing codes did not fundamentally alter the agreement with questionnaire cases. Finally, since the number of participants below 50 years was low for the last wave, we could not evaluate the validity of administrative cases in this age range.

## Conclusion

Our study is the first to estimate the validity of administrative cases of depression in a general population, both of working age and retired. In countries with universal healthcare systems, administrative data offer nation-wide coverage, are more cost-effective to obtain than questionnaire data and can be acquired over a long period of time. In the large cohort analyzed here, administrative cases captured a different dimension of depression than did CIDI-SF cases. Our results suggest that neither of these data sources is superior to the other in the context of large epidemiological studies aiming to identify and quantify risk factors for depression. For future studies with administrative data, we recommend informing the proportion of missing diagnostic codes. We also encourage researchers in the field to verify the validity of cases in a subsample of the population by comparison with a clinical interview.

## Supplementary Information

**Additional file 1.**

## Data Availability

The data that support the findings of this study are available from RAMQ, but restrictions apply to the availability of these data, which were used under license for the current study, and so are not publicly available. Data are however available from the authors upon reasonable request and with permission of RAMQ.

## Abbreviation

CI: Confidence interval
CIDI-SF: Composite international diagnostic interview – short form
DSM-IV: Diagnostic and statistical manual of mental disorders
ICD: International classification of diseases and health problems
IPCW: Inverse probability of censure weighting
NPV: Negative predictive value
PPV: Positive predictive value
PROQ: Prospective Québec
RAMQ: Régie de l’assurance maladie du Québec
RR: Relative risk
WHO: World health organization
