# Supplementary material for: Validation of case definitions of depression derived from administrative data against the CIDI-SF as reference standard: results from the PROspective Québec (PROQ) study
Source: BMC Psychiatry. 2021 Oct 7;21:491. doi: 10.1186/s12888-021-03501-x (PMC8496029; doi:10.1186/s12888-021-03501-x)
Supplement: Supplementary file 1 — Additional file 1. [file 12888_2021_3501_MOESM1_ESM.docx]

**Suppl. Table 1. Numbers of participants classified as administrative and as CIDI-SF cases of depression.**

|  | **Questionnaire cases (CIDI-SF)** | | | | | |
| --- | --- | --- | --- | --- | --- | --- |
| **Administrative cases** | Men | | Women | | All | |
|  | + | - | + | - | + | - |
| **+** Definition A^a^ | 13 | 30 | 28 | 54 | 41 | 84 |
| **-** Definition A^a^ | 63 | 2722 | 117 | 2460 | 180 | 5182 |
| **+** Definition B^b^ | 18 | 59 | 35 | 95 | 53 | 154 |
| **-** Definition B^b^ | 58 | 2693 | 110 | 2419 | 168 | 5112 |
| **Total** | 76 | 2752 | 145 | 2514 | 221 | 5266 |

^a^ Any hospital stay or two medical consultations within the year preceding CIDI-SF.

^b^ Any stay or medical consultation within the year preceding CIDI-SF.

**Suppl. Table 2A. Agreement between administrative and questionnaire cases of depression at T_3_ (2015-2018), stratified by known risk factors.**

| **Sensitivity**  **(CI 95%)** | **Specificity**  **(CI 95%)** | **PPV**  **(CI 95%)** | **NPV**  **(CI 95%)** | κ **(CI 95%)** |  | **Sensitivity**  **(CI 95%)** | **Specificity**  **(CI 95%)** | **PPV**  **(CI 95%)** | **NPV**  **(CI 95%)** | κ  **(CI 95%)** |
| --- | --- | --- | --- | --- | --- | --- | --- | --- | --- | --- |
| Men | | | | |  | Women | | | | |
| 17.1  9.4 – 27.5 | 98.9  98.4 – 99.3 | 30.2  17.2 – 46.1 | 97.7  97.1 – 98.3 | .203  .103 - .303 |  | 19.3  13.2 – 26.7 | 97.9  97.2 – 98.4 | 34.1  24.0 – 45.4 | 95.5  94.6 – 96.2 | .216  .140 - .291 |
| Without university degree | | | | |  | University degree | | | | |
| 19.7  13.3 – 27.5 | 98.1  97.5 – 98.5 | 32.1  22.2 – 43.4 | 96.3  95.6 – 97.0 | .218  .140 - .295 |  | 17.2  10.0 – 26.8 | 98.8  98.3 – 99.2 | 34.1  20.5 – 49.9 | 97.1  96.3 – 97.7 | .211  .114 - .307 |
| ≤ 58 years | | | | |  | >58 years | | | | |
| 23.0  13.2 – 35.5 | 97.3  96.2 – 98.2 | 31.8  18.6 – 47.6 | 95.8  94.5 – 96.9 | .233  .119 - .347 |  | 16.9  11.4 – 23.6 | 98.7  98.3 – 99.0 | 33.3  23.2 – 44.7 | 96.9  96.3 – 97.4 | .204  .133 - .275 |

The prevalence of *administrative cases* (definition A: any hospital stay or two medical consultations within the preceding year) was compared to *CIDI-SF cases* as reference.

**Suppl. Table 2B. Agreement between administrative and questionnaire cases of depression at T_3_ (2015-2018), stratified by known risk factors.**

| **Sensitivity**  **(CI 95%)** | **Specificity**  **(CI 95%)** | **PPV**  **(CI 95%)** | **NPV**  **(CI 95%)** | κ **(CI 95%)** |  | **Sensitivity**  **(CI 95%)** | **Specificity**  **(CI 95%)** | **PPV**  **(CI 95%)** | **NPV**  **(CI 95%)** | κ **(CI 95%)** |
| --- | --- | --- | --- | --- | --- | --- | --- | --- | --- | --- |
| Men | | | | |  | Women | | | | |
| 23.7  14.7 – 34.8 | 97.9  97.2 – 98.4 | 23.4  14.5 – 34.4 | 97.9  97.3 – 98.4 | .214  .123 - .305 |  | 24.1  17.4 – 31.9 | 96.2  95.4 – 96.9 | 26.9  19.5 – 37.4 | 95.7  94.8 – 96.4 | .214  .144 - .284 |
| Without university degree | | | | |  | University degree | | | | |
| 26.5  19.2 – 34.9 | 96.5  95.8 – 97.2 | 26.3  19.1 – 34.7 | 96.6  95.8 – 97.2 | .230  .158 - .302 |  | 20.7  12.7 – 30.7 | 97.7  97.0 – 98.2 | 24.3  15.1 – 35.7 | 97.2  96.4 – 97.8 | .198  .111 - .285 |
| ≤ 58 years | | | | |  | >58 years | | | | |
| 26.2  15.8 – 39.1 | 96.0  94.6 – 97.0 | 26.2  15.8 – 39.1 | 96.0  94.6 – 97.0 | .222  .116 - .328 |  | 23.1  16.8 – 30.4 | 97.4  96.8 – 97.8 | 27.3  18.5 – 33.2 | 97.0  96.5 – 97.5 | .214  .149 - .279 |

The prevalence of *administrative cases* (definition B: any hospital stay or medical consultations within the preceding year) was compared to *CIDI-SF cases* as reference.

**Suppl. Table 3. Agreement between additional definitions of administrative and questionnaire cases of depression at T_3_ (2015-2018).**

|  | **Sensitivity**  **(CI 95%)** | **Specificity**  **(CI 95%)** | **PPV**  **(CI 95%)** | **NPV**  **(CI 95%)** | κ  **(CI 95%)** |
| --- | --- | --- | --- | --- | --- |
| Definition A of administrative cases extended to 18 months ^a,c^ | 20.8  15.7 – 26.8 | 97.9  97.5 – 98.3 | 29.9  22.8 – 37.8 | 96.7  96. – 97.2 | .220  .161 – .268 |
| Definition B of administrative cases extended to 18 months ^b,c^ | 27.6  21.8 – 34.0 | 96.1  95.5 – 96.6 | 22.9  18.0 – 28.5 | 96.9  96.4 – 97.4 | .216  .164 –.268 |
| Definition A of administrative cases extended to 2 years ^a,d^ | 24.3  18.6 – 30.7 | 97.7  97.3 – 98.1 | 30.3  23.4 – 37.9 | 96.9  96.4 – 97.4 | .243  .183 –.304 |
| Definition B of administrative cases extended to 2 years ^b,d^ | 31.1  24.8 – 37.9 | 95.5  94.9 – 96.0 | 21.8  17.2 – 27.0 | 97.1  96.6 – 97.6 | .221  .169 – .273 |
| Definition B of administrative cases excluding controls with missing codes ^b,e^ | 32.1  25.1 – 39.8 | 96.3  95.7 – 96.8 | 25.6  19.8 – 33.1 | 97.3  96.7 –97.7 | .253  .191 – .315 |
| Definition B *vs.* stem question for depression in CIDI-SF ^b^ | 18.7  15.3 – 22.5 | 97.6  97.1 – 98.0 | 41.9  35.2 – 48.9 | 92.8  92.1 – 93.5 | .218  .174 – .262 |
| Definition B restricted to the following codes: 296.2, 296.3, 300.4, 311; F32, F33 ^b^ | 14.0  9.7 – 18.3 | 98.5  98.2 – 98.8 | 28.7  20.4 – 38.2 | 96.5  95.9 – 96.9 | .166  .109 – .224 |
| Definition B if diagnosed by mental health specialists ^b^ | 3.6  1.6 – 7.0 | 99.6  99.4 – 99.8 | 29.6  13.8 – 50.2 | 96.1  95.6 – 96.6 | .056  .015 – .098 |

Unless otherwise stated, the reference is CIDI-SF cases of depression dichotomized at DSM-IV criteria (5 symptoms of depression), and the administrative cases include all codes in Suppl. Table 4 registered during the indicated period preceding measurement of CIDI-SF. ^a^ Any hospital stay or two medical consultations within the indicated period. ^b^ Any hospital stay or medical consultation within the indicated period. ^c^ 18 months preceding measurement of CIDI-SF. ^d^ 18 months preceding + 6 months following measurement of CIDI-SF. ^e^ Participants with any missing code and no known depression-related code were excluded.

**Suppl. Table 4. ICD-9 and ICD-10 codes used to define administrative cases of depression.**

| **ICD-9** | **ICD-10** | **Diagnostic** |
| --- | --- | --- |
| 296.0 |  | Bipolar disorder, single manic episode |
| 296.1 |  | Manic disorder recurrent episode |
| **296.2** |  | **Major depressive disorder, single episode** |
| **296.3** |  | **Major depressive disorder, recurrent** |
| 296.4 |  | Bipolar disorder, most recent episode (or current) manic |
| 296.5 |  | Bipolar disorder, most recent episode (or current) depressed |
| 296.6 |  | Bipolar disorder, most recent episode (or current) mixed |
| 296.7 |  | Bipolar disorder, most recent episode (or current) unspecified |
| 296.8 |  | Other and unspecified bipolar disorders |
| 296.9 |  | Unspecified episodic mood disorder |
| **300.4** |  | **Dysthymic disorder** |
| 309.x |  | Adjustment reaction |
| **311.x** |  | **Depressive disorder, not elsewhere classified** |
|  | F30 | Manic episode |
|  | F31 | Bipolar disorder |
|  | **F32** | **Major depressive disorder, single episode** |
|  | **F33** | **Major depressive disorder, recurrent** |
|  | F34 | Persistent mood [affective] disorders |
|  | F39 | Unspecified mood [affective] disorder |
|  | F43.2 | Adjustment disorder |

**Bold type:** codes used in the supplementary analysis for a restricted definition of x.

**Suppl. Table 5. One-year prevalence of depression and known-groups analysis for sex, age, education level and psychological distress excluding controls with missing codes.**

|  | **CIDI-SF cases** | | | **Administrative cases ^a,b^** | | |
| --- | --- | --- | --- | --- | --- | --- |
|  | **Prev.**  **(non-exposed)** | **Prev.**  **(exposed)** | **Ratio**  **(IC95%)** | **Prev.**  **(non-exposed)** | **Prev.**  **(exposed)** | **Ratio**  **(IC95%)** |
| **Total** | 3.83% | |  | 4.81% | |  |
| **Sex**  **(Ref. male)** | 2.56% | 5.18% | 2.02  1.48 - 2.77 | 3.46% | 6.24% | 1.80  1.37 – 2.37 |
| **Age**  **(Ref. >58 years old** | 3.52% | 5.12% | 1.45  1.04 – 2.04 | 4.21% | 7.26% | 1.72  1.29 – 2.30 |
| **Education T_1_ (Ref. univ. degree)** | 3.40% | 4.15% | 1.22  0.90 - 1.66 | 3.81% | 5.68% | 1.49  1.13 – 1.97 |
| **Psychological distress T_2_**  **(Ref. < 26.2)** | 2.74% | 7.21% | 2.63  1.93 - 3.59 | 3.78% | 8.31% | 2.20  1.66 – 2.90 |

^a^ Participants with any missing code and no known depression-related code were excluded. ^b^ Definition B of administrative cases: any hospital stay or medical consultation within the year
